# Pathways to myeloproliferative neoplasm presentation and time to diagnosis: results from a cross-sectional study

**DOI:** 10.3399/BJGPO.2024.0068

**Published:** 2025-01-15

**Authors:** Emma-Louise Tarburn, Lisa Iversen, Charlotte Robertson, Charlene McShane, Andrew Duncombe, Mary-Frances McMullin, Claire Harrison, Ruben Mesa, Lesley A Anderson

**Affiliations:** 1 Aberdeen Centre for Health Data Science, School of Medicine, Medical Sciences and Nutrition, University of Aberdeen, Aberdeen, UK; 2 School of Medicine, Medical Sciences and Nutrition, University of Aberdeen, Aberdeen, UK; 3 Haematology Department, Aberdeen Royal Infirmary, Aberdeen, UK; 4 Centre for Public Health, School of Medicine, Dentistry and Biomedical Sciences, Queens University Belfast, Belfast, UK; 5 Haematology Department, University Hospital Southampton NHS Foundation Trust, Southampton, UK; 6 Haematology Department, Guy’s and St Thomas’ NHS Foundation Trust, London, UK; 7 Atrium Health Levine Cancer Institute, Charlotte, NC, US

**Keywords:** cancer, neoplasms, diagnosis, general practice

## Abstract

**Background:**

Early cancer recognition is key to improving patient outcomes. Diagnosis is often delayed in patients with myeloproliferative neoplasms (MPNs), putting them at risk of thromboembolic events and other complications pre-diagnosis. A clear understanding of the barriers to presentation and diagnosis is required.

**Aim:**

To explore barriers and factors influencing delayed presentation and diagnosis of MPNs.

**Design & setting:**

A cross-sectional study of patients with MPN within the UK and the Republic of Ireland.

**Method:**

An online cross-sectional survey of patients with MPN was undertaken. Symptoms and factors influencing patient and GP delay were examined. Adjusted odds ratios (aORs) were calculated to explore the relationship between these factors and patient and GP delay.

**Results:**

Most (80.2%) of the 620 patients completing the survey reported symptomatic presentation. The most common symptoms associated with patient delay were pruritus (aOR 1.89, 95% confidence interval [CI] = 1.19 to 3.01), headaches (aOR 1.86, 95% CI = 1.13 to 2.82), and concentration difficulties (aOR 1.75, 95% CI = 1.12 to 2.76). Attributing symptoms to ageing (aOR 1.92, 95% CI = 1.19 to 3.11) and not wanting to burden the GP (2.04, 95% CI = 1.24 to 3.39) were significantly associated with patient delay. Those reporting >3 blood cancer warning signs were more likely to experience GP delay than those experiencing fewer (aOR 3.26**,** 95% CI = 1.75 to 6.29), and lack of relational continuity of GP care was significantly associated with GP delay (aOR 3.41, 95% CI = 1.65 to 7.28).

**Conclusion:**

Debunking misconceptions around ageing, encouraging timely communication with GPs, and improving relational continuity of GP care could assist in reducing diagnostic delays, prevent potentially fatal disease complications, and ultimately improve outcomes for patients with MPN.

## How this fits in

Early cancer presentation and detection is vital to improving prognosis, yet the broad symptom profile of haematological malignancies make timely diagnosis difficult. The current study explores risk factors for delayed presentation and diagnosis of the myeloproliferative neoplasms (MPNs), a rare group of blood cancers that put patients at risk of life-threatening thromboembolic events. To our knowledge, this is the first study to assess the complexities in the decision to present or refer in this group of cancers alone and highlights the significance of barriers to help-seeking, unawareness of blood cancer warning signs, and relational continuity of GP care in delays to diagnosis.

## Introduction

The classic MPNs polycythaemia vera (PV), essential thrombocythaemia (ET), and primary myelofibrosis (PMF) are uncommon haematological malignancies recognised for their debilitating symptom profiles and genomic instability.^1^ Incidence rates for PV, ET, and PMF vary between males and females. For ET, there is a higher incidence in females with an estimated ratio of 1.47:1; however, males are more likely to be diagnosed with PV (1.19:1) and PMF (2:1).^2^ While it is unknown what aetiological factors drive clinical MPNs, mutations that directly or indirectly promote cytokine-independent upregulation of the Janus kinase/signal transducer and activator of transcription pathway commonly affect patients with PV, ET, and PMF.^3–8^ The result is proliferation, marrow fibrosis, organomegalies, thrombohaemorrhagic events, and constitutional symptoms.^9^

Collectively, the haematological malignancies are the third largest cause of cancer-related mortality within the UK,^10^ with 41 000 individuals diagnosed annually.^10^ They represent one in 10 cancer cases in the Republic of Ireland (RoI).^11^ While early presentation, diagnosis, and treatment are key determinants of cancer survival,^12^ nearly one-third of patients with blood cancer are diagnosed via emergency presentation and experience a lower 3-year survival rate (40%) compared with GP-diagnosed cases (77%).^13^

Countries with 'gatekeeper' primary care systems, such as the UK, also experience poorer cancer outcomes than those where patients directly access specialist care.^14^ Improving early presentation and diagnosis of blood cancers requires understanding the decisions to present and refer to identify opportunities for early intervention.^15^

Frameworks for early intervention^16,17^ have reduced the diagnostic interval for several cancers.^18^ However, the time from initial presentation to referral, diagnosis, and treatment of the haematological malignancies remains lengthy. Even patients with more prevalent haematological malignancy subtypes are likely to experience multiple GP appointments before referral.^18^

A UK-based study estimated that the MPNs have the longest overall time to diagnosis of all haematological cancers,^19^ supporting more recent evidence suggesting delayed MPN diagnosis is common.^20^ These delays put patients with MPN at risk of cerebrovascular accidents, myocardial infarction, and other thrombotic events before diagnosis,^21^ which are reduced after disease management strategies start. Therefore, prompt presentation, recognition, and diagnosis are imperative to ensure the wellbeing of patients with MPN. The MyeloprolIferative Neoplasms: RoUTEs to DiagnosiS (MINUTES) Survey was conducted to understand barriers and factors influencing patient and GP-related delay in patients with MPN within the UK and RoI.

## Method

### Questionnaire development

A literature review identified no published, validated tools capturing the information required for the study and informed the development of the MINUTES instrument. The instrument was designed as an online survey using the REDCap electronic data capture tool hosted by the University of Aberdeen^22,23^ and was reviewed by an expert panel to ensure appropriate data capture. The instrument was piloted then revised before data collection.

### Patient and public involvement

Patient representatives from patient charity MPN Voice, the MyelOproliferative neoplasmS: An In-depth Case Control (MOSAICC) study, and NHS sites were involved in the study design and survey items, and advised on patient materials. Patient representatives were members of the steering committee, which advised on data analysis, interpretation, and dissemination of findings. The Aberdeen Centre for Health Data Science patient and public involvement group provided feedback on the questionnaire comprehensibility and readability.

### Data collection

A voluntary, cross-sectional survey of patients with MPN within the UK and RoI was disseminated online during October 2022. Study invitations were issued via a social media flyer on MPN Voice webpages (>4000 followers at dissemination) and by an email containing a weblink to the survey distributed to MPN Voice email subscribers (*n* = 3102). To be eligible to participate, individuals who accessed the weblink were asked to confirm that they: 1) had a clinically confirmed MPN diagnosis; 2) were resident within the UK or RoI; 3) were an English-language speaker; 4) were aged ≥18 years; and 5) did not have cognitive decline. Individuals who did not meet these inclusion criteria were unable to complete the survey.

### Questionnaire content

The final questionnaire was based on the constructs of Walter *et al*’s Model of Pathways to Treatment.^24,25^ The recommendations for reporting instrument development were also followed.^26^ The instrument comprised of four sections consisting mainly of closed questions with pre-defined response options. Few required qualitative responses in free-text fields. Only data from quantitative responses are reported here.

The questionnaire (see Supplementary Information S1) took 20–25 minutes to complete. Data collected included demographic (age group, sex, ethnicity, and country of residence), socioeconomic (household income, educational attainment, and employment status), and presentation type (symptomatic versus incidental) information.

Skip logic was used to divert participants to separate questions tailored to their diagnostic route. Responders with symptomatic presentation answered items (*n* = 7) about symptoms experienced, barriers and facilitators to presentation, and the time from first symptom occurrence to GP presentation. Those reporting incidental diagnosis were asked items (*n* = 4) about the event(s) that led to their diagnosis. A list of MPN-related symptoms was provided and responders asked whether they had experienced any in the year before diagnosis. Responders were asked about the number of GP appointments and number of different GPs seen before referral.

### Data analysis

Demographic information was described using counts and percentages. Demographic and disease differences between incidental and symptomatic cases were investigated (by χ^2^). Difference in symptoms reported before diagnosis by disease subtype was investigated (by χ^2^). Patient delay (from first symptom to presentation) was categorised as short (<12 weeks) or long (≥12 weeks), and GP delay (from initial presentation to referral) was categorised as short (<6 months) or long (≥6 months), in line with a previous study.^15^ The relationship between the COVID-19 pandemic and delay was investigated (by χ^2^). Symptoms (*n* = 23) were assessed individually, by frequency, and as counts for blood cancer warning signs^27^ (fatigue, night sweats, unexpected weight loss, pruritus, satiety, and bruising and/or bleeding). Adjusted odds ratios (aORs) and associated 95% confidence intervals (CIs) for risk factors, barriers to presentation and diagnosis, initial consultation outcome (diagnosed with another condition, no action taken, and tests ordered versus referred to a specialist), and facilitators of early presentation were calculated using logistic regression. Models controlled for MPN subtype, age at survey, biological sex, educational attainment, employment status, marital status, smoking, and alcohol consumption, all factors that were assumed to potentially contribute to delay. Only complete cases were analysed. Statistical analyses were conducted using R (version 4.3.1).

## Results

### Drop-off

**Figure 1.**
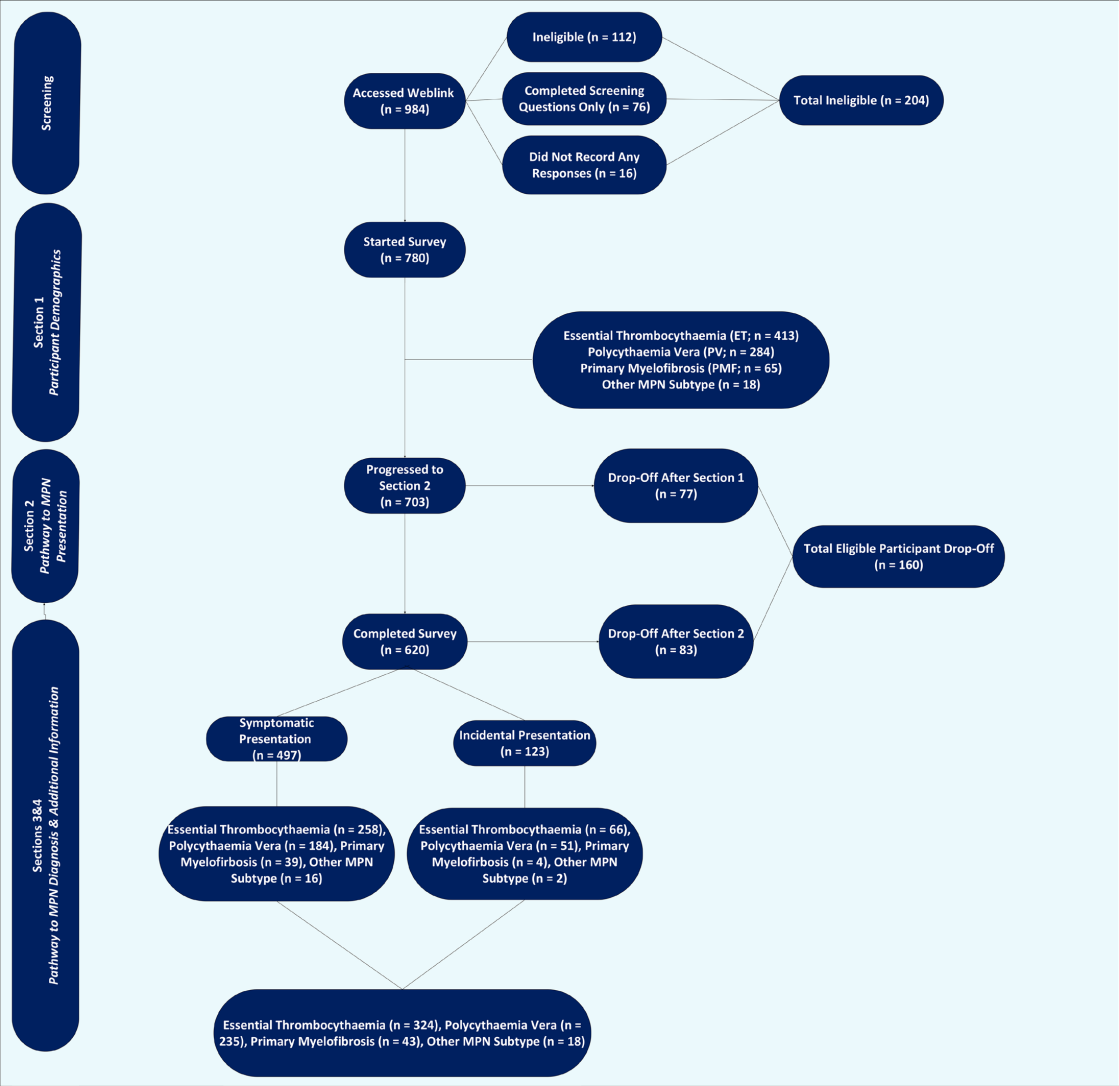
Flow diagram of survey participation. ET = essential thrombocythaemia. MPN = myeloproliferative neoplasm. PMF = primary myelofibrosis. PV = polycythaemia vera.

Owing to the survey distribution methods, it was not possible to calculate a response rate. Of the 780 eligible participants who accessed the survey (Figure 1), 620 completed it, giving a 20.5% drop-off rate. There was a significant difference in the drop-off proportion by MPN subtype (PMF = 33.9%, ET = 22.0%, and PV = 16.9%; *P* = 0.008).

### Participants

Most responders were female (*n* = 459/620, 74.0%; see Supplementary Table S1). The majority reported ET (*n* = 324, 52.3%) followed by PV (*n* = 235, 37.9%), PMF (*n* = 43, 6.9%), and 'Other' (*n* = 18, 2.9%). The majority of participants reported symptomatic presentation to a GP for MPN related symptoms (n = 497, 80.2%). There were no differences observed between incidental and symptomatic cases by demographic or disease characteristics, or between MPN subtype and patient or GP delay (χ^2^ test; *P*>0.05) (data not shown). Little variation was observed between reported symptoms and MPN subtype for the symptomatic cases, with the exception of erythema (more prevalent in patients with PV; χ^2^, *P*<0.001), pruritus (PV; *χ^2^, P*<0.00001), weight loss (PMF; *χ^2^*, *P*<0.0001) and bruising and/or bleeding (PMF; *χ^2^*, *P*<0.05). A marked overlap in symptom type and frequency between the incidental and symptomatic cases was observed.

### Barriers to and facilitators of patient delay

The most common reason for delayed presentation was waiting for symptoms to alleviate; however, this finding was not associated with delay (42.7%; aOR 1.38, 95% CI = 0.88 to 2.17); Table 1. Thinking symptoms or health-related changes were related to age was associated with delay (aOR 1.92, 95% CI = 1.19 to 3.11) as was not wanting to burden the GP (aOR 2.04**,** 95% CI = 1.24 to 3.39). Participants were half as likely to delay (aOR 0.52, 95% CI = 0.32 to 0.83) once a family member or friend had expressed concern about their symptoms or health-related changes.

**Table 1.** Facilitators of, barriers to, and time to presentation for patients with MPN waiting ≥12 weeks to present ('long', *n* = 176) versus those who presented sooner ('short', *n* = 218)

Category	*n* (%)	Proportion waiting ≥12 weeks before presentation, *n*/*N* (%)	Unadjusted OR (95% CI)	Adjusted OR (95% CI)^a^
**Facilitator of short presentation**				
Concerned something was seriously wrong	83 (16.7)	40/137 (29.2)	0.92 (0.55 to 1.54)	0.91 (0.52 to 1.59)
Symptoms or health-related changes were getting worse	208 (41.9)	35/171 (20.5)	1.07 (0.56 to 2.02)	0.90 (0.52 to 1.44)
Symptoms or health-related changes were not getting better	245 (49.3)	108/169 (63.9)	0.88 (0.57 to 1.35)	0.85 (0.54 to 1.33)
Wanted reassurance from doctor	234 (47.1)	100/163 (61.3)	0.76 (0.49 to 1.17)	0.73 (0.46 to 1.15)
A family member or friend expressed concern about symptoms or health-related changes	140 (28.2)	55/164 (33.5)	0.61 (0.39 to 0.94)	0.52 (0.32 to 0.83)
Worried after reading information that symptoms or health-related changes were serious	44 (8.9)	21/147 (14.3)	1.07 (0.56 to 2.02)	1.07 (0.53 to 2.15)
Symptoms or health-related changes started to impact on quality of life	232 (46.7)	104/168 (61.9)	0.91 (0.60 to 1.40)	0.89 (0.56 to 1.43)
**Barrier to delayed presentation**				
Difficulties getting an appointment with a doctor	67 (13.5)	34/170 (20.0)	1.24 (0.73 to 2.12)	1.11 (0.63 to 1.95)
Waiting to get an appointment with preferred doctor or nurse	31 (6.2)	12/158 (7.6)	0.70 (0.32 to 1.47)	0.87 (0.38 to 1.93)
Thought symptoms or health-related changes were related to age	114 (22.9)	64/169 (37.9)	1.84 (1.18 to 2.88)	1.92 (1.19 to 3.11)
Thought symptoms or health-related changes were related to another pre-existing medical condition	97 (19.5)	51/166 (30.7)	1.46 (0.91 to 2.33)	1.39 (0.84 to 2.30)
Did not want to burden or waste the time of your doctor or nurse	96 (19.3)	58/170 (34.1)	2.17 (1.35 to 3.50)	2.04 (1.24 to 3.39)
Could not get, or were waiting for a face-to-face appointment	42 (8.5)	16/157 (10.2)	0.70 (0.36 to 1.35)	0.60 (0.28 to 1.21)
Symptoms or health-related changes did not cause any concern or did not think they were very serious	96 (19.3)	43/164 (26.2)	0.92 (0.57 to 1.47)	0.93 (0.56 to 1.56)
Were worried or afraid that you may get a serious diagnosis	80 (16.1)	35/171 (20.5)	0.92 (0.55 to 1.50)	0.90 (0.52 to 1.54)
Were worried doctor would not take concerns seriously	92 (18.5)	47/173 (27.2)	1.29 (0.81 to 2.08)	1.35 (0.80 to 2.27)
Hoped or was waiting for symptoms to alleviate	212 (42.7)	105/169 (62.1)	1.44 (0.95 to 2.19)	1.38 (0.88 to 2.17)

^a^Adjusted for myeloproliferative neoplasm subtype, sex, age group, marital status, education level, employment status, pre-tax income, current smoker, currently consumes more than the recommended weekly alcohol allowance. MPN = myeloproliferative neoplasm. OR = odds ratio.

**Figure 2.**
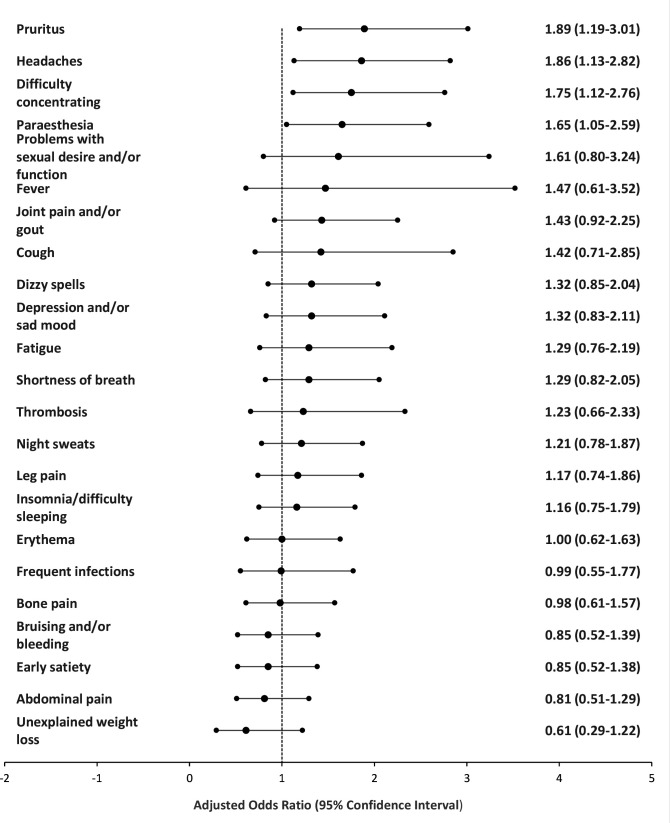
Symptons and time to presentation for patients with myeloproliferative neoplasms (MPNs) taking ≥12 weeks ('long', *n* = 218) from first symptom onset to presentation versus those who presented <12 weeks from first symptoms onset to presentation ('short', *n* = 176). Adjusted for MPN subtype, sex, age group, pre-tax household income, education level, and marital status. Symptoms presented are based on those in the validation MPN-Symptoms Assessment Form.^26,27^

Several symptoms were associated with greater odds of patient delay (pruritus [aOR 1.89, 95% CI = 1.19 to 3.01], headaches [aOR 1.86, 95% CI = 1.13 to 2.82], paresthesia [aOR 1.65, 95% CI = 1.05 to 2.59], and concentration difficulties [aOR 1.75, 95% CI = 1.12 to 2.76]) after adjusting for confounding variables (Figure 2).

**Figure 3.**
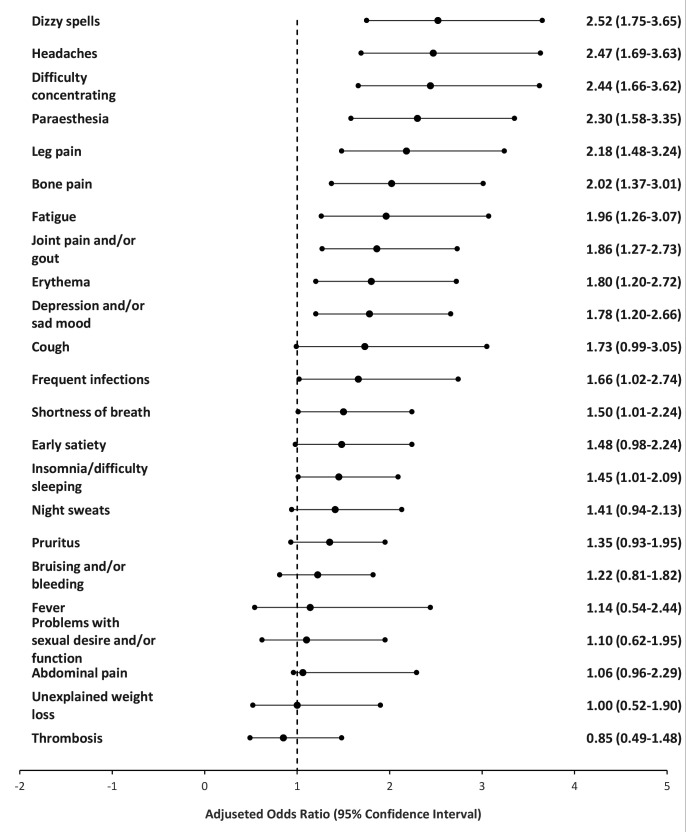
Symptoms and time to diagosis for patients with myeloproliferative neoplasms (MPNs) taking ≥6 months ('long', *n* = 273) from initial presentation to referral ('short', *n* = 287). Adjusted for MPN subtype, sex, age group, pre-tax household income, education level, marital status, current alcohol consumption, and smoker status. Symptoms presented are based on those in the validated MPN-Symptoms Assessment Form.^26,27^

The chances of patient delay increased with symptom count, with those reporting ≥7 symptoms before diagnosis significantly more likely to experience patient delay than those reporting <7 (Table 2). This was also observed in those reporting blood cancer warning signs,^27^ with those reporting three warning signs three times as likely to experience patient delay than those reporting no or fewer (aOR 3.24, 95% CI = 1.44 to 7.60).

**Table 2.** Number of symptoms experienced by patients with MPN taking ≥12 weeks ('long', *n* = 176) from symptom onset to presentation versus those who presented sooner ('short', *n* = 218)

Category	*n* (%)	Unadjusted OR (95% CI)	Adjusted OR (95% CI
**Symptoms, *n*** **^b^**			
<3	88 (17.7)	1.00	1.00
4–6	117 (23.5)	1.81 (0.94 to 3.58)	1.84 (0.94 to 3.70)
7–10	131 (26.4)	2.42 (1.26 to 4.78)	2.37 (1.21 to 4.75)
>10	161 (32.4)	2.55 (1.37 to 4.88)	2.52 (1.33 to 4.93)
**Blood cancer warning signs, *n*** **^c^**			
0	59 (11.9)	1.00	1.00
1	110 (22.1)	2.44 (1.14 to 5.47)	2.36 (1.09 to 5.39)
2	129 (26.0)	2.94 (1.41 to 6.49)	2.87 (1.35 to 6.42)
3	104 (20.9)	3.25 (1.50 to 7.41)	3.24 (1.44 to 7.60)
>3	96 (19.3)	2.28 (1.06 to 5.15)	2.13 (0.95 to 4.96)

^a^Adjusted for myeloproliferative neoplasm subtype, sex, age group, current smoking status, marital status, education level, employment status, pre-tax income and current alcohol intake. ^b^Symptoms are based on those in the validated MPN-Symptom Assessment Form.^30,61^ ^c^Blood cancer warning signs:^27^ fatigue, night sweats, pruritus, satiety, bruising and/or bleeding, and unexpected weight loss. MPN = myeloproliferative neoplasm. OR = odds ratio.

### Risk factors to GP delay

In total, 10 of 23 symptoms assessed were significantly associated with GP delay (dizzy spells, headaches, difficulty concentrating, paraesthesia, leg pain, bone pain, fatigue, joint pain and/or gout, erythrema and depression and/or sad mood; Figure 3). No symptoms were associated with a short time to diagnosis.

Lack of relational continuity of GP care (having to see ≥4 GPs before referral) was associated with GP delay (aOR 3.41, 95% CI = 1.65 to 7.28, *P*<0.001). The number of GP consultations preceding referral was associated with GP delay (Table 3).

**Table 3.** Risk factors and time to diagnosis for patients with MPN taking >6 months ('long', *n* = 273) from symptom onset to diagnosis versus those who were diagnosed sooner ('short', *n* = 287)

Risk factor	*n*	Proportion reporting 'long' time to diagnosis, *n*/*N* (%)	Unadjusted OR (95% CI)	Adjusted OR (95% CI)^a^
Number of appointments for MPN-related symptoms before referral^b^	1	37/145 (25.5)	1.00	1.00
	2–3	82/185 (44.3)	2.32 (1.46 to 3.76)	2.29 (1.35 to 3.94)
	≥4	129/168 (76.8)	9.65 (5.82 to 16.40)	7.38 (4.02 to 13.90)
Number of different GPs seen for MPN-related symptoms before referral	1	63/182 (34.6)	1.00	1.00
	2–3	130/277 (46.9)	1.67 (1.14 to 2.47)	1.20 (0.72 to 2.00)
	≥4	80/101 (79.2)	7.20 (4.14 to 13.00)	3.41 (1.65 to 7.28)

^a^Adjusted for myeloproliferative neoplasm subtype, sex, age group, pre-tax household income, education level, marital status, current alcohol consumption, and smoker status. ^b^Symptoms are based on those in the validated MPN-Symptom Assessment Form.^30,61^ MPN = myeloproliferative neoplasm. OR = odds ratio.

Symptom count was associated with GP delay, with those reporting ≥10 symptoms before diagnosis more likely to experience GP delay than those reporting ≤3 (aOR 5.49, 95% CI = 2.91 to 10.80) (Table 4). This relationship was also seen for blood cancer warning signs, with those reporting >3 warning signs >3 times more likely to experience GP delay than those experiencing no or one warning sign (aOR 3.26, 95% CI = 1.75 to 6.29). Of those reporting incidental diagnoses, 34/123 reported >3 warning signs in the year prior to diagnosis (data not shown).

**Table 4.** Number of symptoms experienced in patients with MPN taking >6 months ('long', *n* = 273) from symptom onset to diagnosis versus those who were diagnosed sooner ('short', *n* = 287)

Category	*n* (%)	Unadjusted OR (95% CI)	Adjusted OR (95% CI)^a^
**Symptoms, *n* ^b^**			
≤3	204 (32.9)	1.00	1.00
4–6	184 (29.7)	1.64 (1.05 to 2.55)	1.58 (1.00 to 2.51)
7–9	161 (26.0)	2.82 (1.81 to 4.45)	2.78 (1.75 to 4.46)
≥10	71 (11.5)	6.17 (3.34 to 11.9)	5.49 (2.91 to 10.80)
**Blood cancer warning signs, *n* ^c^**			
0–1	88 (14.2)	1.00	1.00
2	142 (22.9)	2.37 (1.25 to 4.66)	2.13 (1.10 to 4.26)
3	158 (25.5)	3.37 (1.81 to 6.51)	3.01 (1.59 to 5.92)
>3	232 (37.4)	3.58 (1.98 to 6.75)	3.26 (1.75 to 6.29)

^a^Adjusted for myeloproliferative neoplasm subtype, sex, age group, employment status, smoking status, current alcohol intake, number of doctors seen before referral and number of GP appointments before referral. ^b^Symptoms are based on those in the validated MPN-Symptom Assessment Form.^30,61^ ^c^Blood cancer warning signs:^27^ fatigue, night sweats, pruritus, satiety, bruising and/or bleeding, and unexpected weight loss. MPN = myeloproliferative neoplasm. OR = odds ratio.

There were no significant associations observed between any of the demographic or disease characteristics examined and GP delay. None of the relationships appeared to be affected by the COVID-19 pandemic (data not shown). Initial clinical outcome was significantly associated with GP delay (diagnosed with another condition [aOR 2.69, 95% CI = 1.25 to 5.94], no action taken or told to come back if symptoms worsen [aOR 3.34, 95% CI = 1.54 to 7.52], and tests ordered [aOR 1.52, 95% CI = 0.93 to 2.50]) compared with referral to a specialist.

## Discussion

### Summary

In the MINUTES study, we found that waiting at least 12 weeks from first symptom onset to presentation was common among patients with MPN, and lack of relational continuity of GP care had a significant impact on timely diagnosis. Lack of knowledge of blood cancer symptoms was observed, with more than one-quarter of incidental cases experiencing >3 blood cancer warning signs in the year before diagnosis. Non-specific symptoms delayed diagnosis by increasing GP delay.

### Strengths and limitations

To our knowledge, this is the first in-depth description of the complex diagnostic pathway in patients with MPN using a unique survey instrument. A previous study assessing time to diagnosis of MPN grouped patients with MPN with other entities and did not estimate the magnitude of risk factors for delay.^19^ The survey instrument design conformed with the Model of Pathways to Treatment^24,25^ and drew on constructs from existing tools assessing barriers to cancer diagnosis.^15,28,29^ This ensured consistency in the definitions and methods used and enables comparison with other studies. Symptoms examined within the instrument were specific to MPN and predetermined from existing literature and consultation with haematologists.^30–32^ All items were reviewed by an expert panel to ensure interpretability, comprehensiveness, and appropriateness of response options.

No differences were found between MPN subgroup for patient or GP delay. The limited sample of responders reporting PMF means small variations between MPN subtypes may have been masked. While biological sex was controlled for in our analyses, females were disproportionally represented in the sample and the results may therefore not accurately reflect the characteristics and perspectives of the MPN population. Female over-representation is a common observation in cross-sectional studies,^33,34^ which some researchers have attributed to difficulties in contacting male participants.^35^ Some evidence also suggests that females are more likely to engage in health-information seeking than males.^36^ Although it is unknown if this had an impact on the current study, it is possible that females were more likely to engage with the charity support group used to target recruitment. Future studies of patients with MPNs should explore approaches, such as targeted recruitment strategies, to encourage male participation.

It is possible that those who self-selected to participate have different characteristics, including symptoms and diagnostic experiences, than those who did not participate, potentially limiting generalisability of the findings. Participants reporting PMF were also less likely to complete the entire survey. PMF is the most clinically severe MPN subtype, and with higher median age at diagnosis than PV and ET.^37^ Although the reason for the low completion rate observed in patients with PMF is unknown, these factors may have impacted survey completion. Further research is required using qualitative interviews to supplement these results, and to help uncover nuances and insights that may be masked using a quantitative approach.

Although adjustments provided insight into the potential effects of relational continuity of GP care, it is evident that a more refined approach is necessary to accurately capture its complexities and effect on GP delay.

Participants were diagnosed at various time intervals before survey completion, which may lead to recall bias. GP consultations and data about symptoms presented at those consultations were not corroborated by medical record review. Further research should incorporate assessment of primary care data and GP perspectives to provide further insights into the complex pathway of MPN diagnosis.

While statistical analyses were adjusted for potential confounding factors, there may be residual confounding through unmeasured or inaccurately measured variables. This may have led to biased estimates of the true effect of factors related to delayed presentation and diagnosis.

### Comparison with existing literature

There were no associations between any demographic variables and patient delay, despite similar studies finding lower educational attainment and low socioeconomic status to be barriers to presentation.^38–40^

Delays to presentation and diagnosis were observed for many symptoms, including pruritus, headaches, paraesthesia, and difficulty concentrating. All are known MPN symptoms and included in validated assessments of MPN symptom burden.^30–32^ A conceptual framework for early cancer diagnosis proposed by Koo *et al* highlights the importance of symptom epidemiology in facilitating prompt cancer diagnosis; however, they acknowledge that a positive predictive value threshold of 3% is required to prioritise patients for fast-tracked investigations.^41^ The classic MPN symptoms fall short of this threshold, and haematological malignancies collectively have broad symptom signatures with low positive predictive value.^42^ There are multiple plausible explanations for symptoms commonly experienced in patients with blood cancer, making the decision to seek help or refer challenging. Our results concur with a comprehensive synthesis of qualitative studies, which highlighted symptom appraisal to be a significant barrier to presentation for multiple cancer sites.^43^ Previous work reported around half of patients with blood cancer delayed help-seeking as they assumed their symptoms would alleviate,^44^ whereas another suggested patients with cancer normalise symptoms; for example, by linking night sweats to menopause.^45^ Symptom normalisation has also been demonstrated to be important in GP delay, with GPs suggesting and/or exploring several explanations to explain blood cancer symptoms.^46^ A 2019 study investigating lymphoma diagnosis found that both patients and GPs failed to appraise presenting symptoms collectively, rather judging each symptom individually.^46^ Similarly, in a rapid review of the causes of delays in blood cancer diagnosis, symptom interpretation was found to be a barrier to prompt diagnosis at both the patient and system level.^47^

Patients who are told to monitor symptoms and return if they persist may delay seeking further care owing to interpreting such instructions as reassurance. This highlights a potential flaw in GP safety-netting procedures, where patients may hesitate to seek additional consultations after being reassured or told their symptoms aren't concerning. This supports previous findings that suggest GP safety-netting procedures may not sufficiently engage patients to return if symptoms do not resolve,^48^ and aligns with those in another study that highlighted the impact of the initial assessment on patients' decisions to re-present to primary care.^49^

Thinking symptoms were related to ageing and not wanting to burden the GP were significant barriers to early presentation. These findings highlight the difficulties patients face in negotiating their eligibility, or candidacy, for healthcare services. The tendency to delay help-seeking owing to perceived concerns around burdening their GP or misattribution of symptoms underscores the complex interplay between individual perceptions and healthcare access.^50,51^ The expectation of age-related decline has been implicated in symptom normalisation and subsequent delayed help-seeking in other haematological malignancies.^46^

Social support was an influential factor in early presentation, with participants reporting concern from family or friends less likely to delay presentation, mirroring similar findings in patients with breast and colorectal cancer.^52^ In contrast, another study reported that some patients delayed help-seeking owing to lack of concern from family or friends.^46^

In the MINUTES study, lack of relational continuity of GP care was associated with GP delay, with an increasing trend observed in the number of different GPs seen pre-referral in those with delayed diagnosis. Through interviewing UK GPs, Green *et al* described the significance of relational continuity of care, with GPs acknowledging that this influences their ability to get to know individuals, thus affecting the investigation of suspected cancer.^53^ This was comparable with the findings from other studies that reported lack of relational continuity of GP care between consultations was a cause of delayed diagnosis.^54,55^ Use of the Candidacy Framework to assess how doctor–patient interactions affected perceptions of patients’ eligibility to access healthcare services highlighted the importance of continuity of care when negotiating eligibility, finding trusting relationships to be a significant theme in the help-seeking decision.^50^

Numerous studies into the impact of primary care deliverance during the COVID-19 pandemic highlighted difficulties faced by patients, such as reduced access, barriers to virtual consultation, and reluctance to attend healthcare settings.^56–58^ A 2021 report by The Health Foundation found that there were 31 million fewer primary care appointments from April 2020–March 2021 compared with the previous year.^59^ Despite this, we did not find any significant differences in time to presentation or diagnosis in those diagnosed before or during the COVID-19 pandemic.

The MINUTES study, which focused primarily on assessing barriers to prompt MPN diagnosis using the Model of Pathways to Treatment,^24,25^ has provided a preliminary insight into the complexities in the MPN diagnostic pathway. However, further work drawing on the constructs of other theoretical frameworks may be required to explore the effect of the GP–patient with MPN relationship, GP perceptions, and patient-related barriers to identify areas for improving time to MPN diagnosis.

### Implications for practice

From a GP perspective, MPNs are rare conditions. Signs and symptoms of the MPNs are shared across the spectrum of haematological malignancies making investigations for blood cancer-related symptoms imperative in the work-up of these conditions.

For patients, lack of symptom awareness and the concern for social propriety by avoiding burdening their GP is a significant factor in delayed presentation. Efforts directed towards debunking misconceptions around ageing and encouraging timely communication with GPs could help reduce diagnostic delays. Our study has shown that the lack of awareness of blood cancer symptoms, even for those presenting with distinct clusters associated with haematological malignancy, impacts timely diagnosis. Despite numerous interventions targeting blood cancer symptom recognition at the patient and primary care level, our findings suggest that these might lack impact, probably owing to the broad and non-specific nature of associated symptoms. For GPs, clusters of worrying blood cancer-related symptoms may trigger multiple investigations, thus lengthening the time to diagnosis.

While the benefits of the 2-week wait and urgent suspected cancer fast-track guidelines have been numerous, alternative methods that allow fast access to secondary care for non-specific but concerning symptoms outlined in the NHS Long Term Plan^60^ may enable improvement in the diagnostic pathway for MPNs and other haematological cancers. It is imperative to increase awareness of the consequences of late MPN diagnosis in the primary care setting to ultimately improve the prognosis of patients with MPN.

## Data

Data access can be sought through submission of a data request to the MINUTES study steering committee by emailing the lead author. If approved a data transfer arrangement will be required.
